# Harnessing Fast Fourier Transform for Rapid Community Travel Distance and Step Estimation in Children with Duchenne Muscular Dystrophy

**DOI:** 10.3390/s25103234

**Published:** 2025-05-21

**Authors:** Erik K. Henricson, Albara Ah Ramli

**Affiliations:** Department of Physical Medicine and Rehabilitation, School of Medicine, University of California, Davis, 1 Shields Ave., Davis, CA 95616, USA; arramli@ucdavis.edu

**Keywords:** Fast Fourier Transform, step frequency, step length estimation, gait analysis, Duchenne muscular dystrophy, wearable sensors, mobile health (mHealth), machine learning, community ambulation, digital biomarkers, inertial measurement unit (IMU)

## Abstract

Accurate estimation of gait characteristics, including step length, step velocity, and travel distance, is critical for assessing mobility in toddlers, children, and teens with Duchenne muscular dystrophy (DMD) and typically developing (TD) peers. This study introduces a novel method leveraging Fast Fourier Transform (FFT)-derived step frequency from a single waist-worn consumer-grade accelerometer to predict gait parameters efficiently. The proposed FFT-based step frequency detection approach, combined with regression-derived stride length estimation, enables precise measurement of temporospatial gait features across various walking and running speeds. Our model, developed from a diverse cohort of children aged 3–16, demonstrated high accuracy in step length estimation ($R^{2}=0.92$, RMSE=0.06 m) using only step frequency and height as inputs. Comparative analysis with ground-truth observations and AI-driven Walk4Me models validated the FFT-based method, showing strong agreement across step count, step frequency, step length, step velocity, and travel distance metrics. The results highlight the feasibility of using widely available mobile devices for gait assessment in real-world settings, offering a scalable solution for monitoring disease progression and mobility changes in individuals with DMD. Future work will focus on refining model performance and expanding applicability to additional movement disorders.

## 1. Introduction

The number of steps taken over time and the length of those steps are fundamental elements of human gait, which together describe both velocity and the distance traveled during locomotion. The relationships between these variables have been studied extensively and form the foundation of modern gait analysis, both for typically developing individuals and those with Duchenne muscular dystrophy (DMD) and other mobility disorders.

A detailed review of early and contemporary work on this topic is beyond the scope of this paper, but we refer the reader to studies by G.A. Dean, Inman et al., and Todd et al. for typical gait, and Sutherland and D’Angelo for DMD-specific characteristics [1,2,3,4,5].

Complex mathematical models exist that describe the contributions of each joint and muscle group to gait, as well as how anatomical and functional disturbances can lead to clinically significant mobility limitations, which affect daily life in a variety of patient populations. At the most basic level, however, the velocity and length of each step influences how far and how quickly we move throughout our daily activities.

Recently, the European Medicines Agency (EMA) has endorsed the 95th percentile of maximal stride velocity over weeks-long periods of assessment as a clinically meaningful outcome measure of community ambulation. This measure is particularly relevant for clinical trials involving people with DMD [6]. The ankle-worn device approved for this measurement is elegant, accurate, and well tolerated, even by young children [7,8,9,10,11,12]. However, it remains a custom, high-cost, laboratory-grade device produced in small quantities, making it difficult and expensive to use in large-scale public health or registry-style studies involving people with DMD.

To address these challenges, we developed methods and software that leverage widely available mobile phones and consumer-level devices to accurately measure steps and estimate step length, walking speed, and travel distance in the everyday lives of individuals with DMD, individuals with other mobility disorders, and typically developing individuals.

In our previous work, we described how we created a dashboard of clinical features extracted from iPhone accelerometer data collected during ambulation using our Walk4Me software suite [13,14,15]. Our tools extract clinically meaningful temporospatial features of gait to support both classical machine learning and deep learning models. These models are capable of differentiating between the gait patterns of children with Duchenne muscular dystrophy (DMD) and those of typically developing peers.

The tools rely on computationally intensive, individually calibrated regression-based models and AI-driven step detection algorithms. The models relate acceleration forces to steps of a given length, providing highly accurate estimates of step counts, frequencies, lengths, velocities, and distances traveled during short-duration clinical tests such as the 10 and 25 m timed walk/run tests, the 100 m run, and the 6 min walk test. While the tools can also be applied to longer bouts of activity, they require significant processing time for such tasks.

To improve the speed and accuracy of feature estimation during longer bouts of community-based travel, we developed a tool that requires only the participant’s height and step frequency as inputs. Inspired by the step length estimation models developed by Dean and Todd [1,3], we created a novel step length prediction tool specifically for DMD children and typical controls. This tool is based on multi-speed step length calibration data from our original cohort. Additionally, we used rapid, FFT-based frequency analysis on time-windowed raw accelerometer data to determine step frequency.

In this paper, we present our equations and techniques, evaluating the accuracy and precision of our estimates by comparing them to both ground-truth observations and estimates generated using our original, individually calibrated methods. Specifically, we introduce a novel FFT-based approach to estimate step frequency, step length, step velocity, and total travel distance using a single waist-worn sensor. We validate our method against ground-truth observations and a machine-learning-based approach (Walk4Me), demonstrating strong agreement. Furthermore, we provide a computationally efficient alternative for large-scale, real-world gait monitoring in children with DMD.

## 2. Experiment-I: Developing an Equation to Predict Stride Length Using Step Frequency and Height in Children with DMD and Typical Controls

### 2.1. Materials and Methods

#### 2.1.1. Assessment of Participants

We conducted short-distance walking events (very slow, slow, self-selected comfortable and fast walks) involving ambulatory children and teens with DMD, as well as age-matched typically developing controls. Assessments were conducted and recorded by a trained clinical evaluator on a marked, straight-line course using previously described methods [16,17,18]. Participants with DMD walked a 10 m distance, while typical controls walked a 25 m course. Participants completed longer-distance, mixed-speed walking and running events including a 100 m walk/run, 6 min walk test, and a self-selected-pace free walk. Assessments were recorded on video for later ground-truth validation of step counts and distances. We recorded triaxial acceleration signals using an iPhone running our Walk4Me application, placed at the waist near the lumbosacral junction as previously described [13,14,15].

We selected the waist, near the lumbosacral region, for accelerometer placement due to its proximity to the body’s center of mass, which provides stable, periodic signals primarily reflecting vertical and forward motion. This location minimizes rotational artifacts and noise compared to limb or upper trunk placements, enhancing signal reliability for accurate step frequency and gait feature extraction across individuals and sessions.

#### 2.1.2. Predictive Modeling of Step Length in DMD Patients and Typical Controls

Using the short-distance walking events, we used mixed-model regression techniques accounting for multiple measures within the participant to explore the relationship between observed step length, step cadence, and standing height in participants with DMD and control participants. We calculated step length as the distance divided by the number of observed steps and cadence as the number of steps per second during each trial. We calculated standing height in meters and included an indicator variable of (1) for individuals with DMD and (0) for controls. Using these inputs, we explored multiple transformations of the raw data to satisfy assumptions of normality in ordinary least squares regression. We then constructed multiple exploratory models to estimate step length based on independent variables of step cadence, height, and DMD status and their combined interactions. We evaluated model performance based on the presence of statistically significant contributing variables and interactions, model $R^{2}$ values, and minimization of error.

### 2.2. Results

#### 2.2.1. Participants

Participant characteristics are shown in Table 1. We conducted 261 short-distance walking events in 19 ambulatory DMD patients and 25 typical controls over 67 visits.

#### 2.2.2. Equation for Step Length Prediction

The best-performing model for step length included coefficients for square root-transformed cadence, inverse square root-transformed height, the DMD status indicator variable, and interaction terms between the transformed cadence, height, and DMD status. The adjusted $R^{2}$ for the resulting model was 0.92 (*p* < 0.0001), with a root mean squared error of 0.06 m, indicating good model performance.

**Figure 1:**
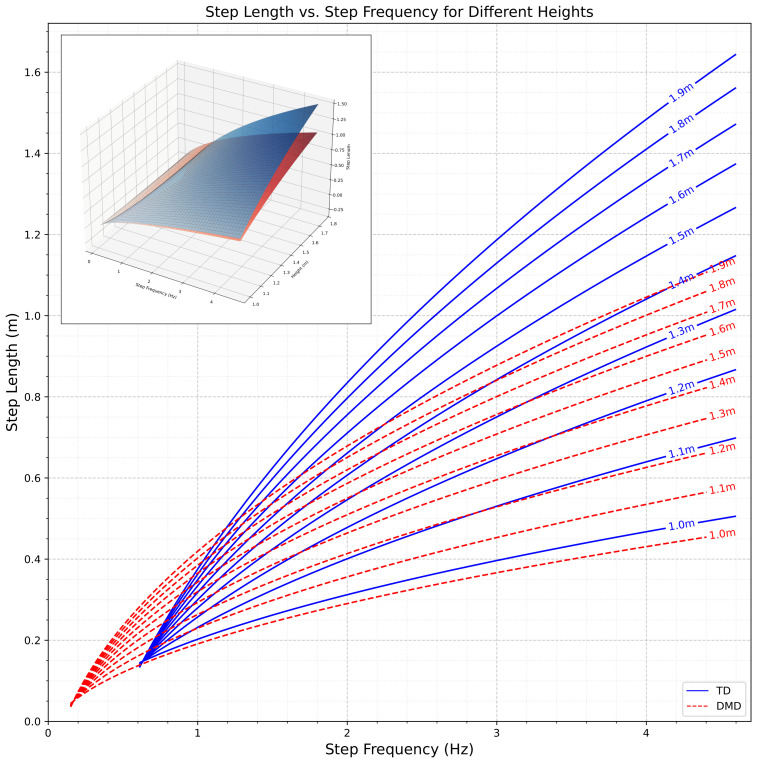
Surface plot illustrating the relationship between step frequency, standing height, and step length in children and adolescents with Duchenne muscular dystrophy (DMD) (red) and typically developing controls (blue). The model is based on Equation (1). The curves illustrates the relationship between step length and step frequency at various heights, as modeled by Equation (1), for TD individuals and those with DMD. The plotted curves represent the regression model, with the blue line depicting TD participants and the red dotted line representing DMD participants.

The model predicting step length (SL) takes a reduced form, where sf is step frequency in steps per second, h is standing height in meters, and DMD(1) serves as the indicator variable (1 = DMD, 0 = Control) that is applied if the individual has a DMD diagnosis. A surface plot of Equation (1) is shown in Figure 1 below.

(1) $$SL\left(sf,h\right)=3.33758\cdot \sqrt{sf}+2.442582\cdot \frac{1}{\sqrt{h}}-3.072612\cdot \left(\sqrt{sf}\cdot \frac{1}{\sqrt{h}}\right)-2.505019+\mathrm{DMD}\left(1\right)\cdot \left(1.87948-1.689478\cdot \sqrt{sf}-1.865428\cdot \frac{1}{\sqrt{h}}+1.664073\cdot \left(\sqrt{sf}\cdot \frac{1}{\sqrt{h}}\right)\right)$$

## 3. Experiment-II: Fast Fourier Transform-Based Estimation of Step Frequency, Step Length, Step Velocity, and Travel Distance from Time-Windowed Raw Accelerometer Data

### 3.1. Materials and Methods

#### 3.1.1. Assessment of Participants

Simultaneously with data collected in Experiment-I (Section 2) and using the same methods, participants completed longer-distance, mixed-speed walking events including a 100 m walk/run, 6 min walk test, and a self-selected-pace free walk.

#### 3.1.2. Fourier-Based Detection of Step Frequency and Count

**Figure 2:**
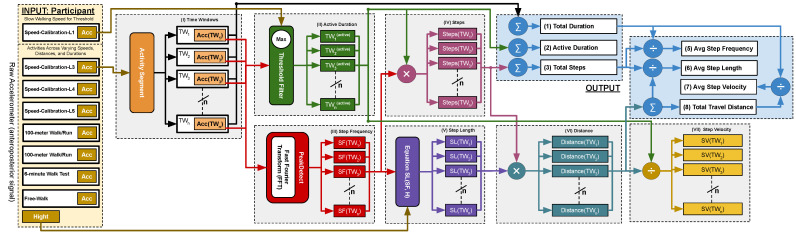
Block diagram illustrating the data flow and processing pipeline of our model for gait analysis. The SC-L1 model dynamically adjusts the threshold, while the input consists of acceleration signals from seven unseen gait activities. Key components of the process include step frequency and height, as well as step length estimation based on Equation (1).

**Figure 3:**
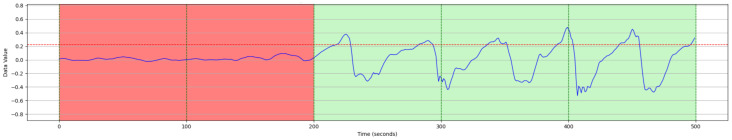
Determination of active and inactive periods based on peak acceleration thresholds in the slowest walking speed calibration (SC-L1).

**Figure 4:**
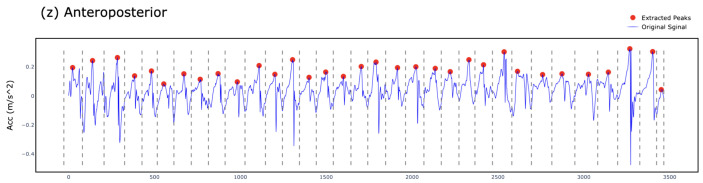
In SC-L1, the raw acceleration signal is represented by the blue time-series, while the red dots indicate the highest peak in each step. The ${\mathrm{peak}}_{i}$ represents the maximum acceleration value in the *i*th step, where *m* is the total number of steps in the SC-L1 activity. The gray dotted vertical lines denote the edge duration of each step. These peak values are subsequently used to compute the mean and standard deviation, which serve as the basis for dynamically determining the threshold.

**Figure 5:**
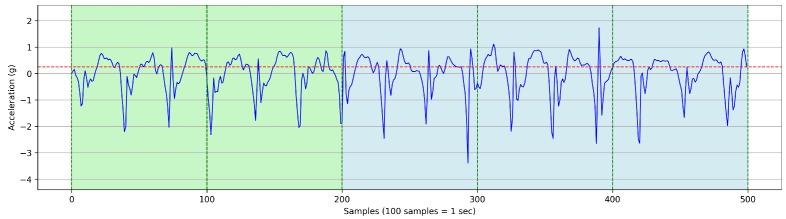
Raw accelerometer data (z-axis) collected during a walking/running trial, demonstrating the time-domain signal collected during ambulation.

**Figure 6:**
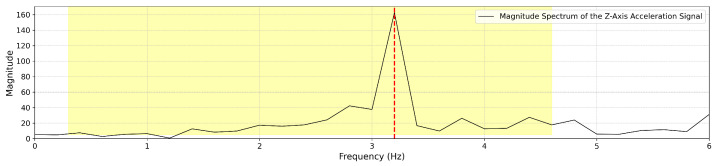
Fast Fourier Transform (FFT) analysis of accelerometer data within a 5 s time window. The figure highlights the dominant step frequency component extracted for gait cycle estimation.

**Figure 7:**
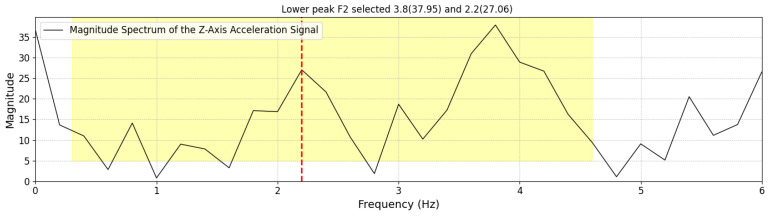
Example of FFT spectrum where the first peak is identified as the step frequency due to its significant magnitude.

**Figure 8:**
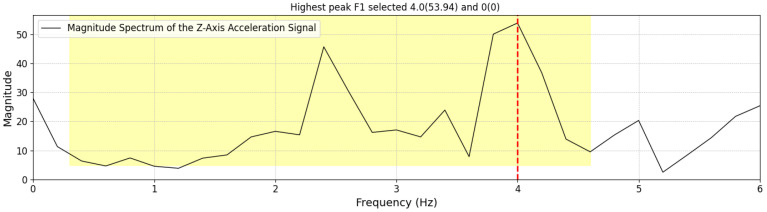
Example of FFT spectrum where the second peak is selected as the step frequency based on predefined criteria.

We developed an iterative process to estimate gait parameters using the anteroposterior (z-axis) raw accelerometer signal as displayed in Figure 2. To ensure reliable frequency-domain analysis, each 5 s time window was preprocessed by removing the mean (detrending) to eliminate baseline drift and applying a bandpass filter to retain signal components between 0.3 and 4.6 Hz. This range captures physiologically relevant step frequencies while suppressing low-frequency drift and high-frequency noise unrelated to gait. These preprocessing steps enhanced the accuracy of the FFT-derived step frequency estimates across all trials. To measure the frequency of steps and estimate the count of steps, we extracted and estimated the following values using these steps:

I **Split the Raw Signal into Time Windows (TWs):**
  We start by collecting the z-axis acceleration data for the activity and then segment the data into 5 s time windows. All time windows (TWs) in this study were fixed at 5 s in duration to ensure consistent frequency-domain analysis. We selected a fixed 5 s non-overlapping time window to balance spectral resolution and sensitivity to gait changes. This duration was empirically chosen based on pilot tests indicating that shorter windows reduced frequency clarity, while longer ones averaged over heterogeneous gait patterns. The 5 s window provided stable, interpretable spectral peaks for both children with DMD and typically developing peers. While the TW length is constant, the amount of active time within each window varies depending on participant movement. This variability in activity levels across TWs affects the number of detected steps and related gait metrics, even though the window size itself does not change. Within each window, we determine the inactive periods for each individual second in the $TW_{i}$.
II **Determine the Active Duration Time in Each TW:**
  We collect z-axis acceleration data at the slowest speed (SC-L1—very slow walking) and compute the threshold, defined as follows:
  $$\mathrm{Threshold}=\mu_{\mathrm{peaks}}+\sigma_{\mathrm{peaks}}$$
  where ${\mathrm{peak}}_{i}$ denotes the highest peak in a single step at index *i*, with the number of steps in SC-L1 ranging from i=1 to *m*.
  $$\begin{array}{ccc} \mu_{\mathrm{peaks}} & =\frac{1}{m}\sum_{i=1}^{m}{\mathrm{peak}}_{i}\;\mathrm{and}\;\sigma_{\mathrm{peaks}} & =\sqrt{\frac{1}{m}\sum_{i=1}^{m}{\left({\mathrm{peak}}_{i}-\mu_{\mathrm{peaks}}\right)}^{2}} \end{array}$$
  This threshold, represented by the red horizontal line in Figure 3, is computed using the mean (μ) and standard deviation (σ) of peak values in **SC-L1** as shown in Figure 4. If the maximum G-force in any 1 s period within the TW is greater than this threshold, that second is classified as **active time** (highlighted in green). Otherwise, it is classified as **inactive time** (highlighted in red). This classification rule is applied to every second within the TW.
  $$\begin{array}{c} {\mathrm{TW}}_{i}^{\left(\mathrm{active}\right)}=\left\{\begin{array}{cc} {\mathrm{TW}}_{i}, & \mathrm{if}\;\max[\mathrm{Acc}({\mathrm{TW}}_{i})]\geq \mathrm{Threshold}\\ 0, & \mathrm{otherwise} \end{array}\right. \end{array}$$
III **Estimate Step Frequency per TW:**
  For each TW, we convert the z-axis acceleration signal (Figure 5) to the frequency domain. Figure 6 illustrates a frequency spectrum (FFT) outcome for the raw TW. We use the highest peak in the frequency domain to determine the step frequency for each TW, denoted as $\mathrm{Step}\;{\mathrm{Frequency}}_{\mathrm{TW}}$.
  $$\mathrm{Step}\;{\mathrm{Frequency}}_{{\mathrm{TW}}_{i}}=\mathrm{PeakDetect}\left(\mathrm{FFT}[\mathrm{Acc}({\mathrm{TW}}_{i})]\right)$$
  where
  - ${\mathrm{Acc}}_{\mathrm{TW}}$ is the raw acceleration signal recorded within a given TW.
  - FFT(·) applies the Fast Fourier Transform (FFT) to convert the time-domain acceleration signal into the frequency domain. For example, Figure 6 illustrates the frequency domain representation of the TW shown in Figure 5.
  - PeakDetect(·) identifies the dominant peak frequency from the transformed signal, corresponding to the step frequency in that TW.
  - $\mathrm{Step}\;{\mathrm{Frequency}}_{\mathrm{TW}}$ is the estimated step frequency for the given time window.
  In the frequency domain, we identify the highest peak (by magnitude), which represents the step frequency of the selected TW. The following constraints are applied during detection:
  (a) Identify the highest peak in the frequency domain within the range of 0.3 to 4.6 Hz; otherwise, the step frequency is set to 0. The frequency range of 0.3 to 4.6 Hz was chosen based on empirical observations of functional gait cadence in our study population. The lower bound (0.3 Hz) was selected to exclude non-locomotor movements such as positional adjustments, while the upper bound (4.6 Hz) reflects the fastest step cadences observed in teenage athletes during running tasks in a separate pilot study involving high-performing youth, which is beyond the scope of this analysis.
  (b) If two peaks exist and the lower-frequency peak is less than 60% of the highest frequency peak, but its magnitude is at least 60% of the highest frequency peak, then the smaller peak is selected as the $\mathrm{Step}\;{\mathrm{Frequency}}_{\mathrm{TW}}$ as shown in Figure 7. Otherwise, the highest frequency peak is determined as the $\mathrm{Step}\;{\mathrm{Frequency}}_{\mathrm{TW}}$ as shown in Figure 8. In some cases, particularly during running or advanced Trendelenburg-style gaits with a pronounced lateral component, we observed harmonic patterns in the frequency spectrum in which a higher-magnitude peak appeared at approximately twice the actual step frequency.
  Once the $\mathrm{Step}\;{\mathrm{Frequency}}_{\mathrm{TW}}$ for a given TW is determined, we calculate the following per TW:
IV **Estimate the Number of Steps per TW**: Multiply the step frequency by the active duration time (as discussed in Section 3.1.2 item II) within the 5 s time window (0–5 s).
  $${\mathrm{Steps}}_{{\mathrm{TW}}_{i}}=\mathrm{Step}\;{\mathrm{Frequency}}_{TW_{i}}\times \mathrm{Active}\;{\mathrm{Duration}}_{TW_{i}}$$
V **Estimate Step Length per TW**: Estimate the step length for each TW using Equation (1). This calculation is based on the participant’s height and the step frequency derived from the FFT-based step frequency peaks:
  $$\begin{array}{c} \mathrm{Step}\;{\mathrm{Length}}_{{\mathrm{TW}}_{i}}=SL\left(SF_{{\mathrm{TW}}_{i}},H\right) \end{array}$$
  where SL(sf,h) is defined in Equation (1), $sf_{\mathrm{TW}}$ is the step frequency for the given time window, and *h* is the participant’s standing height.
VI **Estimate the Travel Distance per TW**: Compute the distance for each TW using the following:
  $$\begin{array}{c} {\mathrm{Distance}}_{{\mathrm{TW}}_{i}}={\mathrm{Steps}}_{TW_{i}}\times \mathrm{Step}\;{\mathrm{Length}}_{TW_{i}} \end{array}$$
VII **Estimate Step Velocity per TW**: Calculate the step velocity in meters per second for each TW. The step velocity for each TW is estimated using the following:
  $$\begin{array}{c} \mathrm{Step}\;{\mathrm{Velocity}}_{{\mathrm{TW}}_{i}}=\frac{{\mathrm{Distance}}_{{\mathrm{TW}}_{i}}}{{\mathrm{TW}}_{i}^{\left(\mathrm{active}\right)}} \end{array}$$

Then, we use the calculated values per TW to estimate the total values:

1. **Estimate the Total Duration**: Sum the ${\mathrm{TW}}_{i}$ across all time windows (TWs) using the following:
  $$\begin{array}{c} \mathrm{Total}\;\mathrm{Duration}=\sum_{i=1}^{n}{\mathrm{TW}}_{i} \end{array}$$
  where *n* represents the total number of time windows (TWs), and ${\mathrm{TW}}_{i}$ denotes the duration of the *n*-th time window.
2. **Estimate the Active Duration**: Sum the Active Duration ${\mathrm{TW}}_{i}^{\left(\mathrm{active}\right)}$ across all time windows (TWs) using the following:
  $$\begin{array}{c} \mathrm{Active}\;\mathrm{Duration}=\sum_{i=1}^{n}{\mathrm{TW}}_{i}^{\left(\mathrm{active}\right)} \end{array}$$
3. **Estimate the Total Number of Steps**: Sum the number of steps across all TWs using the following:
  $$\begin{array}{c} \mathrm{Total}\;\mathrm{Steps}=\sum_{i=1}^{n}{\mathrm{Steps}}_{{\mathrm{TW}}_{i}} \end{array}$$
  where *N* is the total number of time windows, and ${\mathrm{Steps}}_{\mathrm{TW}}$ represents the number of steps detected in each individual time window.
4. **Estimate Average Step Frequency**: Compute the average step frequency using the following:
  $$\begin{array}{c} \mathrm{Avg}\;\mathrm{Step}\;\mathrm{Frequency}=\frac{\mathrm{Total}\;\mathrm{Steps}}{\mathrm{Total}\;\mathrm{Duration}} \end{array}$$
  In this analysis, we used total duration (including both active and inactive periods) when calculating average step frequency. This decision aligns with our ground-truth reference, which was based on total steps divided by total time during each bout. While this provides a representative average for controlled clinical tasks (e.g., the 6 min walk test), we acknowledge that daily ambulation is discontinuous. In future work, particularly in real-world monitoring contexts, we plan to compute average step frequency using only the active duration to better reflect instantaneous gait performance.
5. **Estimate Average Step Length**: Calculate the average step length using the following:
  $$\begin{array}{c} \mathrm{Avg}\;\mathrm{Step}\;\mathrm{Length}=\frac{\mathrm{Total}\;\mathrm{Travel}\;\mathrm{Distance}}{\mathrm{Total}\;\mathrm{Steps}} \end{array}$$
6. **Estimate Average Step Velocity**: Compute the average step velocity using the following:
  $$\begin{array}{c} \mathrm{Avg}\;\mathrm{Step}\;\mathrm{Velocity}=\frac{\mathrm{Total}\;\mathrm{Travel}\;\mathrm{Distance}}{\mathrm{Total}\;\mathrm{Duration}} \end{array}$$
  In this study, we calculated average step velocity using total duration (including both active and inactive periods) to reflect overall community-level mobility. This approach is intended to capture real-world functional performance, where walking is often intermittent and influenced by fatigue, rest breaks, or pauses. For example, a child with DMD may walk for 30 s and then rest for 30 s; the inclusion of rest time provides a more holistic view of daily functional capacity. While active-time velocity is more appropriate for evaluating pure gait performance, our focus here was on mobility density (a clinically meaningful indicator of functional ambulation in community settings).
7. **Estimate Total Travel Distance**: Sum the travel distances of all TWs to estimate the total travel distance for the entire effort using the following:
  $$\begin{array}{c} \mathrm{Total}\;\mathrm{Travel}\;\mathrm{Distance}=\sum_{i=1}^{n}{\mathrm{Distance}}_{{\mathrm{TW}}_{i}} \end{array}$$

#### 3.1.3. Machine Learning-Based Prediction of Gait Characteristics Using the Walk4Me System

Accurate and efficient estimation of gait characteristics is essential for mobility assessment and clinical evaluation. The Walk4Me system integrates machine learning (ML) methodologies to predict gait parameters from accelerometer signals obtained via a single waist-worn device. This section provides an overview of our ML-based approach, which has been described in detail in our prior publications [13,14,15], and is included here for comparative purposes. While our primary focus in this study is the application of FFT-based analysis due to its suitability for high-volume data processing, the ML approach remains relevant for understanding gait characteristics.

**Overview of Machine Learning Approach**: Our ML-based method employs supervised learning techniques to estimate key gait parameters, including step count, step length, stride duration, and gait speed. Raw accelerometer signals are preprocessed through feature extraction, followed by classification and regression modeling. The primary workflow includes the following:

- **Preprocessing and Feature Engineering:** raw accelerometer data undergo filtering and transformation to extract temporal and frequency-domain features.
- **Gait Event Detection:** a deep learning-based classifier is utilized to identify gait events, leveraging annotated datasets for model training.
- **Step Length Estimation:** regression models map peak acceleration values to estimated step lengths using individualized calibration procedures.
- **Distance and Speed Prediction:** by aggregating estimated step lengths and step durations, the system calculates gait speed and total distance traveled.

**Comparative Analysis with FFT-Based Methods**: In prior studies, the ML-based approach demonstrated high accuracy in step detection and gait characterization, particularly in individuals with atypical gait patterns, such as those with Duchenne muscular dystrophy (DMD) [14]. However, FFT-based methods, which focus on spectral decomposition of accelerometer signals, offer advantages in processing efficiency, particularly for large-scale data streams. By converting time-series accelerometer signals into frequency components, FFT enables rapid identification of dominant gait-related frequencies, making it well suited for automated and high-throughput analysis.

While the Walk4Me system’s ML-based approach has been validated in clinical settings and remains a viable option for gait characterization, FFT methods provide computational efficiency and scalability, particularly for handling high-volume data. The inclusion of ML-based methods in this study serves as a benchmark for comparison, highlighting the trade-offs between data-driven prediction models and frequency-domain analysis.

#### 3.1.4. Evaluation of Precision and Bias of Model-Based Estimates Compared to Ground-Truth Observation

We compared the step count, step frequency, step length, step velocity, and travel distance estimates from the FFA and Walk4Me models to observed measurements. Our validation data included a variety of walking tasks, such as comfortable and fast walks over 10 m, 25 m, and 100 m, a 6 min walk test, and a free walk. We compared the FFT model and Walk4Me models to the observations, as well as the two models against each other. To assess agreement and bias, we used Bland–Altman analysis to calculate mean percentage differences and limits of agreement (Supplementary Materials). Since walking is cyclical, we normalized residuals and limits of agreement to the percentage of each measurement to account for error increasing proportionally with repetition. We also compared model slopes and intercepts using Passing–Bablok regression, with strong agreement defined as a confidence interval including 1 for slopes and 0 for intercepts [19]. For acceptable agreement, we chose slopes between between 0.9 and 1.1 and intercepts within 2% of the maximum observed ground-truth value.

Additionally, we calculated Lin’s Concordance Correlation Coefficient ($\rho_{c}$) to assess both precision and bias, with strong agreement >0.95 and a lower limit of 0.9 for acceptable agreement [20].

We replicated methods described by Kirk and colleagues [21], who used mean absolute error (MAE) and mean relative absolute error (MRAE) to describe the accuracy of step velocity measurements during structured and daily-living activities. Anticipating that distributions of absolute error would be non-normal, we instead compared median absolute error (MdAE) and median absolute percent error (MdAPE) values and interquartile ranges for absolute and percent error of each model in typical controls, DMD participants, and the combined population for step count, step frequency, step length, step velocity, and travel distance.

### 3.2. Results

#### 3.2.1. Participants

Participant characteristics are identical to Experiment-I (Section 2) and are shown in Table 1. We conducted 325 ambulatory task assessments in 19 participants with DMD and 25 typically developing controls. All tasks included ground-truth total distances and elapsed times. Video recordings required for calculation of step counts, step lengths, and step velocities were available for 298 (91%) of the assessments.

#### 3.2.2. Precision and Bias of Estimatred Gait Parameters

**Figure 9:**
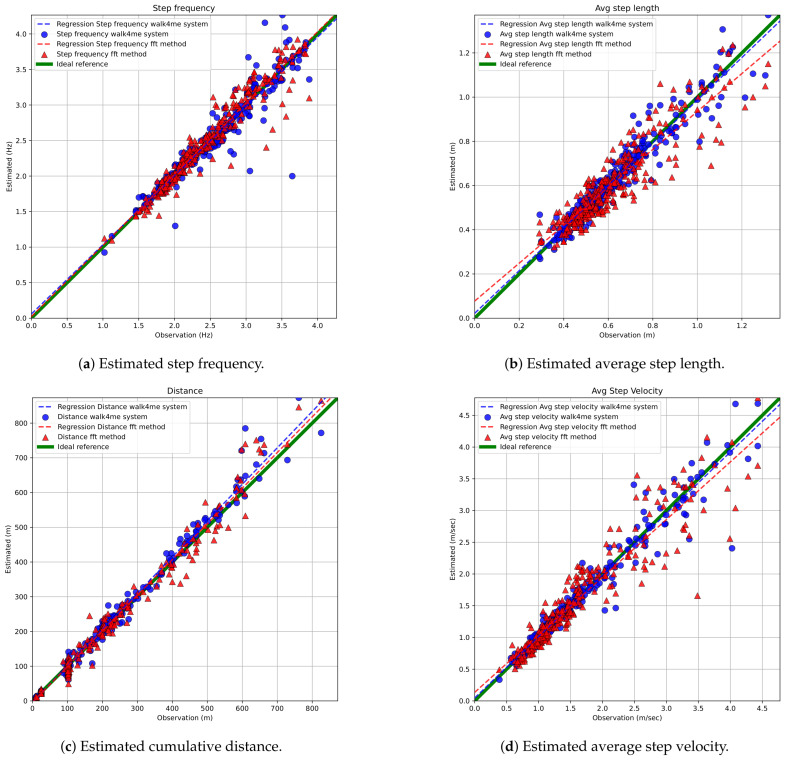
Error analysis for estimated gait parameters across various activities.

**Figure 10:**
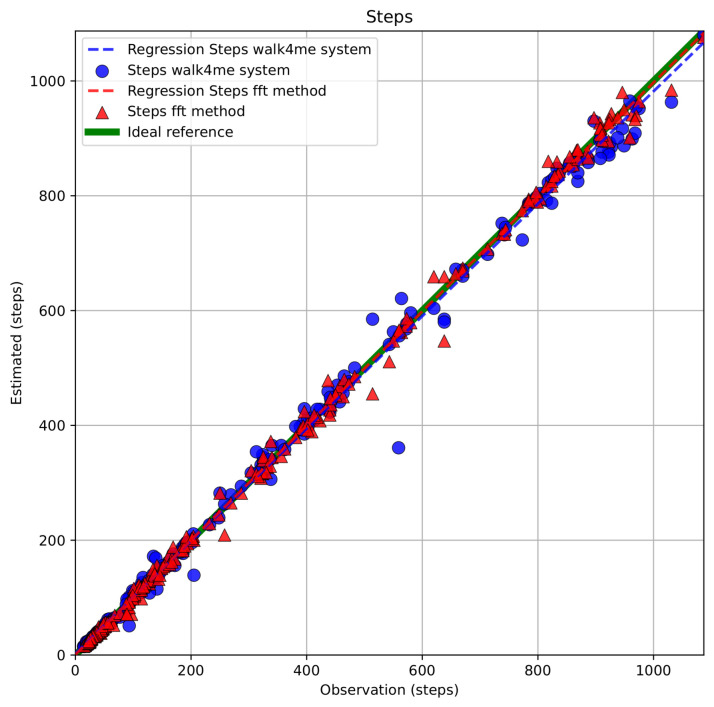
Comparison of estimated step counts using FFT-based and Walk4Me models versus ground-truth step counts.

FFT-based and Walk4Me-based estimates of step count, step frequency, step length, step velocity, and travel distance vs. observed ground-truth measurements are shown in Figure 9 and Figure 10. Results of BA, PB, and LCC are shown comparing each parameter to observed ground-truth measures in Table 2, Table 3 and Table 4. Bland-Altman plots comparing FFT methods to ground-truth measures are provided in the Supplementary Materials.

- **Step count** comparisons demonstrated excellent agreement with observation and between models, falling within our specified criteria for accuracy, precision, and bias. Bland–Altman regression showed mean percent differences <1% and narrow limits of agreement, while Passing–Bablok regression demonstrated strong agreement in both slopes and intercepts. Lin’s ($\rho_{c}$) values > 0.99 indicated high precision and low bias.
- **Step frequency** comparisons demonstrated excellent agreement with observation and between models, falling within our specified criteria for accuracy, precision, and bias. Mean percent differences were <1%, and limits of agreement were narrow, and there was acceptable to strong agreement in both slopes and intercepts. Lin’s ($\rho_{c}$) values between 0.93 and 0.96 indicated moderate to high precision and correspondingly low bias.
- **Step length** estimate comparisons demonstrated strong agreement with observation and between models, falling within our specified criteria for accuracy, precision, and bias. Mean percent differences were <1.29%, and limits of agreement were slightly wider due to the use of group-derived and individually calibrated models. There was acceptable to strong agreement in both slopes and intercepts. Lin’s ($\rho_{c}$) values between 0.91 and 0.96 indicated acceptable to high precision and correspondingly low bias.
- **Distance** comparisons demonstrated excellent agreement with observation and between models, falling within our specified criteria for accuracy, precision, and bias. Mean percent differences were <1%, and limits of agreement were slightly wider due to the use of group-derived and individually calibrated models. There was acceptable to strong agreement in both slopes and intercepts. Lin’s ($\rho_{c}$) values > 0.99 indicated high precision and low bias.
- **Step velocity** estimate comparisons demonstrated strong agreement with observation and between models, falling within our specified criteria for accuracy, precision, and bias. Mean percent differences were <1%, and limits of agreement were slightly wider due to the use of group-derived and individually calibrated models. There was acceptable to strong agreement in both slopes and intercepts. Lin’s ($\rho_{c}$) values between 0.93 and 0.98 indicated moderate to high precision and correspondingly low bias.

We evaluated the overall error of the estimates by assessing the mean absolute error (MAE) for stride velocity measurements across all activities. The FFT models showed an MAE (SD) of [0.17 (0.22) m/s], while the Walk4Me models had an MAE of [0.1 (0.18) m/s]. Our results were consistent with those reported by Kirk and colleagues for complex daily activities.

A comparison of absolute and percentage errors for each metric confirmed that the differences between model-based estimates and ground-truth measurements were not normally distributed (Shapiro–Wilk < 0.0001). As a result, we present the remaining findings using the median and interquartile ranges for the Median Absolute Error (MdAE) and Median Absolute Percentage Error (MdAPE), as shown in Table 5 and Table 6. Overall, both estimation techniques performed well, showing acceptable levels of error.

The strongest agreement was observed for the directly measured items step count and step frequency. In these cases, MdAPE estimates were consistently less than 5% of the ground truth, with the time-windowed FFT-based methods showing a slight advantage in performance. For calculated metrics, FFT-based estimates of step length, distance, and step velocity also showed good agreement, with MdAPE estimates generally under 10% at walking speeds. Similarly, estimates from the Walk4Me machine learning system outperformed the FFT-based methods, with MdAPE results ranging from 3% to 10%.

While our models generally showed lower error rates in typical controls compared to participants with DMD, this trend did not hold during running tasks. We believe this may be due to the presence of a flight phase in typical running, which introduces greater biomechanical variability and may reduce the accuracy of models trained primarily on walking data. In contrast, children with DMD tend to exhibit “Duchenne jogging” with minimal or no flight phase and a narrower range of running speeds. These gait characteristics may more closely resemble walking dynamics, which our models handle more reliably. We are currently collecting additional data across a wider spectrum of running behaviors in children, teens, and adults to refine our equations for use at higher speeds and with variable gait dynamics.

## 4. Discussion

Here, we present a model-based approach to accurately estimate step length in children aged 3 to 16 years with Duchenne muscular dystrophy and typical controls using only step frequency and standing height. When combined with step counts and elapsed total time, the formula presented here is suitable for creating estimates of travel distance for that time interval. The equation, which we created from observations of step frequency and stride length across a typical range of slow to fast walking speeds in toddlers, children, and teens with Duchenne muscular dystrophy and similarly aged typically developing controls, may also be suitable for use as a predictive equation for DMD and typical populations, and to create contrasts between the two at different stages of growth and development.

We further demonstrate that the Fast Fourier Transformation is a computationally efficient and suitable method to estimate step frequency from time-windowed raw signals from inertial measurement units in commercially available smart phones and other wearable devices during longer-duration recording of daily activities. Daily step activity patterns and step/stride velocity of people with DMD have been well described [8,10,11,12,22,23,24,25,26], but less is known about daily distance traveled, and adding estimates of travel distance and velocity may provide new insights into temporal patterns of habitual activity. The total daily step count decreases only gradually as people with DMD progress in their disease, reducing the utility of this metric as a clinical trial outcome measure. However, the drop in high-frequency and high-velocity steps becomes more apparent with loss of ability. Measurement of the 95th percentile of habitual stride velocity—the speed below which 95 percent of steps occur—in the community using ankle-worn IMUs is well developed and is approved as an outcome measure for clinical trials [7,8,9,10,11,12]. Yet, the velocity of steps or strides alone tells an incomplete story that accounts for neither the length of those steps, nor the distances traveled during daily activities, nor the overall patterns of temporospatial gait characteristics over time.

The absolute error of walking velocity measurements with single IMU-based wearable devices has been reported to increase with the complexity of community walking activities [21]. In a recent report by Kirk and colleagues, they reported an absolute error of 0.11 m/s (range 0.09 to 0.13 m/s) in walking velocity across multiple patient groups and a relative absolute error of 20.3% (range 15.48 to 24.88%) for a combined set of simple and complex gait tasks reflective of real-world walking bouts. Using similar methods, we report similar if slightly improved levels of accuracy, adding a controlled running task in toddlers, children, and teens that demonstrates an acceptable level of accuracy using single, waist-worn IMU devices.

**Figure 11:**
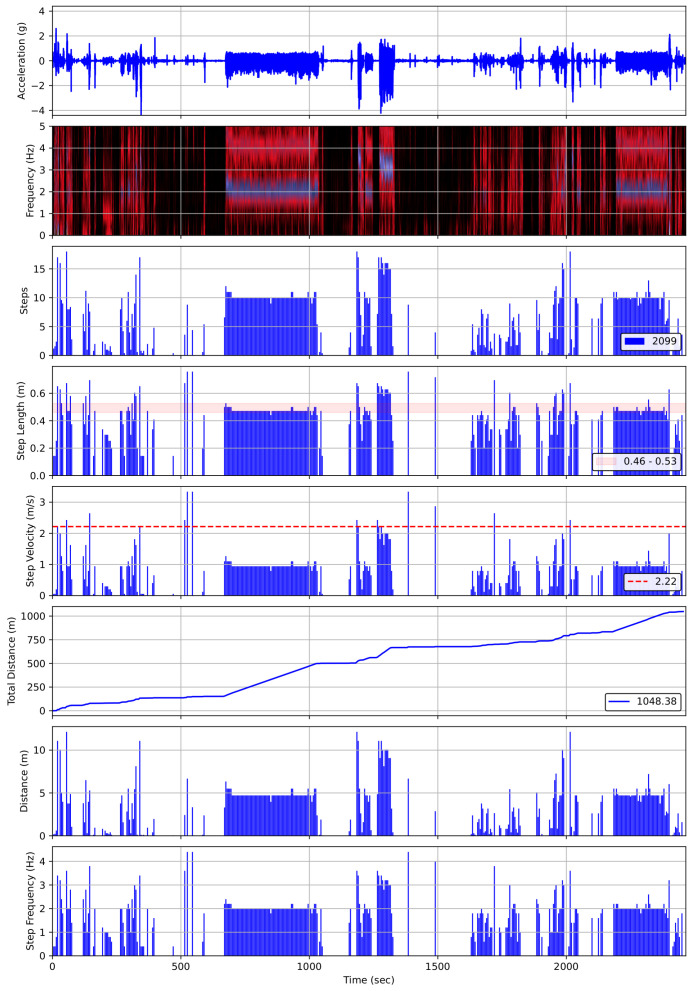
Visualization of time-series gait analysis for an extended activity session, showing frequency, estimated number of steps, estimated step length, step velocity, cumulative travel distance, estimated distance, and step frequency.

Used alone or in combination, our two approaches to IMU data analysis allow us to accurately estimate clinically meaningful gait parameters using a variety of consumer-level, single-sensor devices. The overall agreement between our FFT-based and individually calibrated, AI-based Walk4Me models [13,14,15] indicates that FFT-based estimates can provide an accurate first-pass analysis to identify key events of interest. As illustrated in Figure 11, which depicts data extracted from a 50 min mixed-task activity using FFT-based analysis, and Equation (1), the ability to rapidly generate temporal maps of long-term activity can help us to identify events of interest such as prolonged periods of continuous moderate activity or shorter bouts of high-level burst activity. Here, we display the raw accelerometer signal over time, followed by a time-series FFT visual map of step frequency in 5 s time windows. For each time window, Equation (1) is then used to determine the step length, shown with a band indicating 35–40 percent of the standing height, which is common during self-selected-pace to fast walking. Those time-windowed step length estimates yield average velocities for each step, with the dotted red line indicating the 95th percentile step velocity for the recorded activity. Simultaneously, step count and step length yield estimates of cumulative distance traveled, which ultimately provides the basis for the evaluation of community-based travel over extended periods of time. Notably, the areas of highest step frequency are easily visible highlighted in purple in the time-series FFT panel. These areas represent the 6 min walk test (between approximately 700 s and 1100 s), the 10 m, 25 m, and 100 m walk/run tests (approximately 1200 s to 1400 s), and the “free walk” (approximately 2200 s to 2500 s). In each case, the last panel illustrates both cumulative distance represented on the y-axis and also velocity represented as the slope of the line.

Once such events are identified, we can then employ more computationally intensive AI tools to extract an extended panel of clinically relevant gait features to reflect not only stride length and velocity characteristics, but proportions of forces indicating forward, lateral, and vertical travel that are known to be significantly altered in individuals with a variety of pathological gait patterns. Likewise, the extension of measurement tools into the frequency domain using continuous sensor data and tools like FFT opens up the entire spectral analysis toolbox, enabling us to construct time-series maps of activity that quantitatively and visually combine both the frequency and power of gait events, and allows for the extraction of features from such data for inclusion in future predictive classical machine learning and deep learning AI models.

## 5. Conclusions

This study introduces a novel method for estimating step length, step velocity, and travel distance in children with Duchenne muscular dystrophy (DMD) and typically developing controls using frequency-domain analysis and regression-based modeling. By leveraging Fast Fourier Transform (FFT)-derived step frequency from single, waist-worn consumer-grade accelerometers, our approach enables accurate estimation of gait parameters in real-world settings.

Our results demonstrate that step frequency, when combined with standing height, provides a reliable predictor of step length in both DMD and typically developing children. The proposed model achieves high accuracy (*R*^2^ = 0.92) and low error (RMSE = 0.06 m), validating its applicability in community gait evaluation. Additionally, comparisons between FFT-based methods and AI-driven Walk4Me models indicate strong agreement across multiple gait metrics, reinforcing the reliability of FFT as a computationally efficient alternative for large-scale data processing.

Beyond laboratory settings, this method enhances the feasibility of community-based gait monitoring using widely available consumer devices. The ability to quantify step length, velocity, and travel distance without the need for expensive, custom-built IMUs opens new possibilities for monitoring disease progression in DMD, assessing intervention efficacy, and supporting clinical decision-making in mobility-impaired populations.

Future work will focus on refining our models through larger datasets, expanding analysis to additional movement disorders, and integrating real-time data processing capabilities into mobile health applications. By improving accessibility and scalability, our approach has the potential to contribute significantly to gait assessment and digital health monitoring in both clinical and everyday environments.

## Figures and Tables

**Table 1:** Characteristics of the children included in the study.

Measure	DMD				Control				*p*-Value
	N	Mean	SD	Range	N	Mean	SD	Range	
Age (years)	19	9.79	3.71	(3.0–16.0)	25	7.6	3.33	(2.0–15.0)	0.0273
Height (cm)	19	127.6	14.86	(101.6–153.3)	25	131.36	19.8	(105.5–175.0)	0.6181
Weight (kg)	19	37.19	14.56	(17.2–67.7)	25	33.26	18.01	(18.5–101.0)	0.4070
NSAA Score	17	20.71	7.41	(8.0–32.0)	25	32.96	2.11	(26.0–34.0)	<0.0001
10MRW Time (s)	18	6.44	2.29	(3.66–10.6)	24	3.52	0.83	(2.16–6.05)	<0.0001
6MWT Dis. (m)	18	370.22	84.11	(227.0–519.0)	24	541.69	123.01	(233.0–825.5)	<0.0001

**Table 2:** Precision and accuracy of measurements: FFT method vs. observed (ground truth).

Metric	PB Regression			BA Plot Analysis		Limits of Agreement (%)	Lin’s Concordance Correlation Agreement (%)
	N	Slope (Two-Sided, 95% CI)	Intercept (Two-Sided, 95% CI)	N	Mean % Difference (Two-Sided, 95% CI)		
Step Count	297	1 (0.99, 1)	1 (1, 1.06)	297	0.98 (0.32, 1.63)	−10.22, 12.17	0.9992
Step Frequency (Hz)	298	1.06 (1.03, 1.08)	−0.1 (−0.16, −0.06)	298	0.92 (0.24, 1.59)	−10.68, 12.52	0.9634
Step Length (m)	298	0.99 (0.94, 1.05)	0 (−0.03, 0.03)	298	−1.29 (−2.66, 0.08)	−24.91, 22.32	0.9083
Distance (m)	325	1.03 (1.01, 1.04)	−1.13 (−1.4, −0.35)	325	−0.64 (−2.11, 0.82)	−27, 25.7	0.9917
Step Velocity (m/s)	297	1.05 (1.01, 1.09)	−0.06 (−0.11, −0.01)	297	−0.41 (−1.95, 1.13)	−26.79, 25.97	0.9395

**Table 3:** Precision and accuracy of measurements: Walk4Me system vs. observed (ground truth).

Metric	PB Regression			BA Plot Analysis		Limits of Agreement (%)	Lin’s Concordance Correlation
	N	Slope (Two-Sided, 95% CI)	Intercept (Two-Sided, 95% CI)	N	Mean % Difference (Two-Sided, 95% CI)		
Step Count	297	1 (0.99, 1)	0 (0, 0.2)	297	−0.22 (−1, 0.55)	−13.54, 13.09	0.9979
Step Frequency (Hz)	298	1 (0.99, 1)	0 (0, 0.06)	298	−0.22 (−1, −0.55)	−13.52, 13.08	0.9533
Step Length (m)	296	1.03 (1, 1.06)	−0.02 (−0.03, 0)	296	−0.05 (−0.95, 0.86)	−15.55, 15.46	0.9633
Distance (m)	323	1.04 (1.03, 1.04)	−1.2 (−1.56, −0.86)	323	−0.36 (−1.3, 0.58)	−17.2, 16.48	0.9939
Step Velocity (m/s)	295	1.02 (1, 1.04)	−0.02 (−0.05, 0)	295	−0.12 (−1.06, 0.81)	−16.11, 15.86	0.9754

**Table 4:** Precision and accuracy of measurements: FFT method vs. Walk4Me system.

Metric	PB Regression			BA Plot Analysis		Limits of Agreement (%)	Lin’s Concordance Correlation
	N	Slope (Two-Sided, 95% CI)	Intercept (Two-Sided, 95% CI)	N	Mean % Difference (Two-Sided, 95% CI)		
Step Count	325	1 (0.99, 1)	−1 (−1, 0.08)	325	−0.83 (−1.73, 0.06)	−16.9, 15.24	0.9973
Step Frequency (Hz)	325	0.91 (0.89, 0.93)	0.18 (0.13, 0.22)	325	−0.86 (−1.76, 0.03)	−16.9, 15.21	0.9343
Step Length (m)	323	1.07 (1.02, 1.11)	0.03 (−0.06, 0)	323	1.08 (−0.12, 2.28)	−20.38, 22.54	0.9316
Distance (m)	323	1 (0.99, 1.03)	−0.24 (−0.79, 0.33)	323	0.34 (−1.18, 1.86)	−26.86, 27.54	0.9919
Step Velocity (m/s)	323	0.98 (0.95, 1.02)	0.02 (−0.03, 0.07)	323	0.34 (−1.18, 1.86)	−26.86, 27.53	0.9334

**Table 5:** Median absolute error (MdAE[IQR]) of model-based estimates for activities varying in speed and duration.

**Comfortable/Fast Walk**	Control (n = 82)		DMD (n = 50)		All (n = 132)	
	FFT-based	Walk4Me	FFT-based	Walk4Me	FFT-based	Walk4Me
Step Count (steps)	1 [1–2]	1 [0–1]	1 [1–1]	0 [0–1]	1 [1–2]	0 [0–1]
Step Frequency (Hz)	0.08 [0.03–0.14]	0.05 [0–0.11]	0.11 [0.07–0.15]	0 [0–0.07]	0.09 [0.04–0.14]	0 [0–0.1]
Step Length (m)	0.04 [0.02–0.08]	0.02 [0.01–0.05]	0.05 [0.02–0.08]	0.02 [0.01–0.03]	0.04 [0.02–0.08]	0.02 [0.01–0.04]
Distance (m)	2 [1–4]	0.69 [0.34–1.18]	1 [0.8–2]	0.3 [0.14–0.51]	1 [0.9–3]	0.49 [0.2–1.03]
Step Velocity (m/s)	0.11 [0.04–0.32]	0.04 [0.02–0.07]	0.10 [0.06–0.15]	0.03 [0.01–0.05]	0.11 [0.04–0.23]	0.04 [0.02–0.07]
**Free Walk**	Control (n = 34)		DMD (n = 17)		All (n = 51)	
	FFT-based	Walk4Me	FFT-based	Walk4Me	FFT-based	Walk4Me
Step Count (steps)	10 [4–15]	7.5 [4–16]	5 [3–9.5]	13 [8–21]	6 [4–15]	10 [4–17]
Step Frequency (Hz)	0.05 [0.02–0.08]	0.03 [0.02–0.08]	0.02 [0.02–0.05]	0.05 [0.04–0.08]	0.03 [0.02–0.08]	0.04 [0.02–0.08]
Step Length (m)	0.02 [0.01–0.04]	0.02 [0.01–0.04]	0.04 [0.03–0.09]	0.03 [0.01–0.05]	0.03 [0.01–0.05]	0.02 [0.01–0.04]
Distance (m)	10 [7–15]	9.27 [4.7–14.9]	16 [6–24]	10.5 [5.6–21.8]	10.5 [6.1–23]	9.4 [4.9–17.2]
Step Velocity (m/s)	0.05 [0.03–0.08]	0.04 [0.02–0.08]	0.08 [0.03–0.12]	0.06 [0.03–0.11]	0.06 [0.03–0.09]	0.05 [0.02–0.08]
**6 min Walk Test**	Control (n = 35)		DMD (n = 19)		All (n = 54)	
	FFT-based	Walk4Me	FFT-based	Walk4Me	FFT-based	Walk4Me
Step Count (steps)	9 [5–22]	16 [5–42]	7 [2–11]	10 [1–32]	9 [4–20]	15 [3–37]
Step Frequency (Hz)	0.02 [0.01–0.06]	0.06 [0.01–0.12]	0.02 [0.01–0.03]	0.03 [0–0.09]	0.02 [0.01–0.06]	0.04 [0.01–0.1]
Step Length (m)	0.04 [0.02–0.08]	0.04 [0.02–0.06]	0.02 [0.01–0.04]	0.02 [0.01–0.04]	0.03 [0.01–0.06]	0.03 [0.01–0.06]
Distance (m)	30 [13–61]	23.4 [9.9–41.6]	15 [7–26]	13.8 [6.5–23.7]	24.5 [11–53]	18.4 [8.8–35.4]
Step Velocity (m/s)	0.08 [0.03–0.17]	0.07 [0.03–0.11]	0.04 [0.01–0.07]	0.04 [0.02–0.08]	0.07 [0.03–0.12]	0.06 [0.02–0.11]
**100 m Walk/Run**	Control (n = 41)		DMD (n = 20)		All (n = 61)	
	FFT-based	Walk4Me	FFT-based	Walk4Me	FFT-based	Walk4Me
Step Count (steps)	4 [1–10]	5 [3–9]	3 [2–5]	4 [2–8]	4 [2–7]	5 [2–9]
Step Frequency (Hz)	0.11 [0.04–0.3]	0.13 [0.07–0.31]	0.04 [0.03–0.09]	0.05 [0.04–0.11]	0.09 [0.03–0.18]	0.11 [0.05–0.23]
Step Length (m)	0.07 [0.03–0.17]	0.05 [0.02–0.07]	0.05 [0.01–0.09]	0.03 [0.02–0.05]	0.07 [0.02–0.14]	0.04 [0.02–0.06]
Distance (m)	11 [6–23]	4.9 [2.8–11.9]	9 [3–16]	3.7 [1.9–6.7]	10 [3.3–20]	4.2 [2.2–10]
Step Velocity (m/s)	0.34 [0.14–0.59]	0.12 [0.08–0.37]	0.17 [0.04–0.23]	0.06 [0.03–0.13]	0.22 [0.09–0.51]	0.1 [0.05–0.34]
**All Tasks**	Control (n = 192)		DMD (n = 105)		All (n = 297)	
	FFT-based	Walk4Me	FFT-based	Walk4Me	FFT-based	Walk4Me
Step Count (steps)	3 [1–9]	3 [1–10]	2 [1–5]	1 [0–9]	2 [1–7]	2 [0–10]
Step Frequency (Hz)	0.07 [0.02–0.13]	0.06 [0.01–0.13]	0.07 [0.02–0.13]	0.03 [0–0.09]	0.07 [0.02–0.13]	0.05 [0–0.11]
Step Length (m)	0.04 [0.02–0.08]	0.03 [0.01–0.05]	0.04 [0.02–0.08]	0.02 [0.01–0.04]	0.04 [0.02–0.08]	0.03 [0.01–0.05]
Distance (m)	7 [2–16.5]	2.24 [0.8–13]	3 [1–16]	2.4 [0.35–10]	6 [1.5–16]	3.1 [0.59–11.94]
Step Velocity (m/s)	0.09 [0.04–0.27]	5.9 [2.4–11.3]	0.08 [0.03–0.16]	0.04 [0.02–0.08]	0.09 [0.04–0.21]	0.05 [0.02–0.09]

**Table 6:** Median absolute percent error (MdAPE[IQR]) of model-based estimates for activities varying in speed and duration.

**Comfortable/Fast Walk**	Control (n = 82)		DMD (n = 50)		All (n = 132)	
	FFT-based	Walk4Me	FFT-based	Walk4Me	FFT-based	Walk4Me
Step Count (%)	3.5 [1.5–5.4]	2 [0–4]	4.9 [2–6.7]	0 [0–3.3]	4.3 [1.6–5.6]	0 [0–3.8]
Step Frequency (%)	3.4 [1.5–5.4]	2 [0–4]	5.1 [3.7–6.6]	0 [0–3.3]	4.3 [1.8–5.6]	0 [0–3.8]
Step Length (%)	7.8 [3.5–10.7]	3.8 [1.3–6.9]	10 [4.5–15]	3.1 [1.6–7.1]	8.3 [4–14.7]	3.5 [1.4–6.9]
Distance (%)	8 [4–16]	2.9 [1.4–4.8]	10 [8.1–18.4]	2.8 [1.5–4.9]	10 [4–16]	2.9 [1.4–4.8]
Step Velocity (%)	8 [4–16]	2.9 [1.4–4.8]	10 [8.1–18.4]	2.8 [1.5–4.9]	10 [4–16]	2.9 [1.4–4.9]
**Free Walk**	Control (n = 34)		DMD (n = 16)		All (n = 50)	
	FFT-based	Walk4Me	FFT-based	Walk4Me	FFT-based	Walk4Me
Step Count (%)	2.4 [1.1–4.3]	1.8 [0.7–3.9]	1.2 [0.7–2.4]	3.0 [2.4–6.1]	1.6 [0.9–4.0]	2.5 [1.2–4.0]
Step Frequency (%)	2.4 [1.1–4.3]	1.8 [0.7–3.9]	1.3 [0.8–2.5]	2.5 [2.3–4.5]	1.7 [0.1–4.3]	2.4 [1.1–4.0]
Step Length (%)	5.3 [2.1–7.2]	3.7 [1.7–6.6]	14.5 [5.9–17.9]	5.7 [4.3–10]	6.0 [2.3–10]	4.3 [1.7–8.3]
Distance (%)	4.7 [3.1–7.3]	4.7 [2.2–6.8]	8 [3.1–17.4]	7 [3.4–13]	5.4 [3.1–10.6]	5 [2.7–9.4]
Step Velocity (%)	4.7 [3.1–7.3]	4.2 [1.7–6.8]	9.7 [3.4–18.5]	7.7 [3.6–12.6]	5.2 [3.1–10.5]	4.8 [2.4–8.4]
**6 min Walk Test**	Control (n = 35)		DMD (n = 19)		All (n = 54)	
	FFT-based	Walk4Me	FFT-based	Walk4Me	FFT-based	Walk4Me
Step Count (%)	1.1 [0.6–2.4]	2.3 [0.6–4.5]	0.9 [0.4–1.3]	1.5 [0.1–3.5]	1.0 [0.5–2.2]	2.0 [0.4–2.5]
Step Frequency (%)	1.1 [0.1–2.4]	2.3 [0.6–4.5]	0.9 [0.4–1.4]	1.5 [0.1–3.5]	1.0 [0.6–2.3]	2.0 [0.4–4.5]
Step Length (%)	5.6 [3.1–11]	6 [2.9–10.5]	3.9 [1.9–7.8]	3.9 [1.8–8.8]	5.5 [2.4–10.1]	5.5 [2–10]
Distance (%)	5.4 [2.7–11.1]	4.5 [2–7.6]	3.7 [1.8–7]	3.4 [2.2–6.9]	5.2 [2.2–10]	4.1 [2.2–7]
Step Velocity (%)	5.2 [2.3–10.9]	4.9 [2–7.6]	3.6 [1.2–6.6]	4.2 [1.7–7.8]	5.1 [1.8–9.5]	4.6 [2–7.6]
**100 m Walk/Run**	Control (n = 41)		DMD (n = 20)		All (n = 61)	
	FFT-based	Walk4Me	FFT-based	Walk4Me	FFT-based	Walk4Me
Step Count (%)	3.1 [1.1–8.3]	4.3 [2.1–8.9]	1.5 [1.2–2.8]	2.3 [1.1–3.9]	2.9 [1.1–5.2]	3.5 [1.3–8.6]
Step Frequency (%)	3.1 [1.1–8.3]	4.3 [2.1–8.9]	1.5 [1.2–2.8]	2.3 [1.1–3.9]	2.9 [1.1–5.2]	3.5 [1.3–8.6]
Step Length (%)	8.3 [2.8–20.1]	4.7 [2.9–7.9]	8.2 [1.9–17]	5.5 [3.2–9.1]	8.2 [2.5–19]	4.8 [2.9–8.2]
Distance (%)	10.7 [5.7–22]	4.7 [2.6–11.6]	8.7 [2.9–15.5]	3.5 [1.9–6.6]	9.7 [3.2–19.4]	4.1 [2.2–9.7]
Step Velocity (%)	10.7 [5.7–22.3]	4.7 [2.7–11.6]	8.7 [2.9–15.5]	3.7 [1.9–9.1]	9.7 [3.4–19.4]	4.2 [2.6–10.3]
**All Tasks**	Control (n = 192)		DMD (n = 105)		All (n = 297)	
	FFT-based	Walk4Me	FFT-based	Walk4Me	FFT-based	Walk4Me
Step Count (%)	2.7 [0.9–4.9]	2.4 [0.4–4.5]	2.2 [0.6–5.6]	1.3 [0–3.7]	2.5 [0.9–5.1]	2.1 [0–4.4]
Step Frequency (%)	2.7 [0.9–4.9]	2.4 [0.4–4.5]	2.5 [0.9–5.5]	1.2 [0–3.7]	2.6 [1–5.2]	2.1 [0–4.4]
Step Length (%)	6.3 [2.8–11.6]	4.3 [1.7–7.9]	9.7 [4–15.6]	4.1 [1.7–8.7]	7.1 [3.6–14.7]	4.2 [1.7–8.1]
Distance (%)	6.7 [3.2–15]	3.9 [1.9–6.8]	10 [2.8–14.8]	3.5 [1.8–7.3]	8 [3.1–14.8]	3.7 [1.9–7]
Step Velocity (%)	6.8 [3.2–14.8]	3.8 [1.8–6.7]	10 [2.8–15.5]	3.5 [1.8–7.5]	8 [3.1–15.5]	3.7 [1.8–7]

## Data Availability

Due to the human subject and health information privacy nature of the data and our institutional regulations, we will share information upon presentation of evidence of IRB or ethics board review and completion of appropriate data transfer agreements. Requests for data access can be addressed to the corresponding author.

## Supplementary Materials

A portion of the source code and a demonstration associated with this paper, along with additional results, are available at https://albara.ramli.net/research/ff (accessed on 14 May 2025).
